# Label-Free Microscale Thermophoresis Discriminates Sites and Affinity of Protein–Ligand Binding**

**DOI:** 10.1002/anie.201204268

**Published:** 2012-09-24

**Authors:** Susanne A I Seidel, Christoph J Wienken, Sandra Geissler, Moran Jerabek-Willemsen, Stefan Duhr, Alwin Reiter, Dirk Trauner, Dieter Braun, Philipp Baaske

**Affiliations:** NanoTemper Technologies GmbHFlössergasse 4, 81369 Munich (Germany); Systems Biophysics and Functional NanosystemsLudwig-Maximilians-Universität München, Amalienstrasse 54, 80799 Munich (Germany); Department Chemie und Biochemie and Center of Integrated Protein ScienceLudwig-Maximilians-Universität München, Butenandtstrasse 5–13, Haus F, 81377 Munich (Germany)

**Keywords:** analytical methods, binding affinity, label-free methods, microscale thermophoresis, protein conformation

The analysis of protein binding to small molecules, nucleic acids, and ions not only gives fundamental insights into cellular processes but also paves the way towards improved disease diagnosis and treatment. Herein, we report on a novel label- and preparation-free method to quantify biomolecular interactions and gather additional information on the binding event. The technique is based on the recently developed microscale thermophoresis (MST).

Several approaches to explore biomolecule binding require fluorescent or radioactive labeling.^[1]^ Other methods, such as surface plasmon resonance (SPR) and quartz crystal microbalance (QCM), rely on surface immobilization.^[2]^ But the coupling of a protein to a tag or surface may alter or even inhibit the binding event.^[3]^ Furthermore, the coupling reactions and associated clean-up steps are time consuming and, for some biomolecules, difficult to optimize. This is particularly true for those protein preparations that are typically low in yield or less stable in solution, like membrane receptor systems. A recent solution-based label-free method is the kinetic capillary electrophoresis with mass spectrometry (KCE-MS), which only requires that the binding partners be separable by electrophoresis.^[4]^ Another method is back-scattering interferometry (BSI), which is limited to proteins displaying detectable changes in the refractive index (RI) upon binding and displays remarkable sensitivity for high-affinity interactions.^[5]^ So far, most genuinely label-free studies of protein–ligand interactions have been performed using isothermal titration calorimetry (ITC). ITC provides direct access to the thermodynamic parameters of a binding event but requires considerably high protein concentrations to gain a measurable signal.^[6]^

In contrast MST is characterized by low sample consumption. As the name already implies, it is based on thermophoresis, the directed movement of particles in a temperature gradient.^[8,^^9]^ A temperature difference Δ*T* in space leads to a depletion of the solvated biomolecules in the region of elevated temperature, quantified by the Soret coefficient *S*_T_: *c*_hot_/*c*_cold_=exp(−*S*_T_ Δ*T*).

This thermophoretic depletion depends on the interface between the molecules and the solvent.^[9]^ Under constant buffer conditions, thermophoresis probes the size, charge, and solvation entropy of the molecules. The thermophoresis of a protein typically differs significantly from the thermophoresis of a protein–ligand complex as a result of binding-induced changes in size, charge, and solvation energy.^[10,^^11]^ Even if a binding event does not significantly change the size or charge of a protein, MST can still detect the binding owing to binding- induced changes in the molecules' solvation entropy. Glutamate binding to ionotropic glutamate receptors (iGluRs), for instance, causes a conformational change observable by MST. Binding leads to a closure of the protein’s clamshell-like ligand-binding domain (LBD), inducing receptor activation (Figure 1 B).^[12,^^13]^

**Figure 1:**
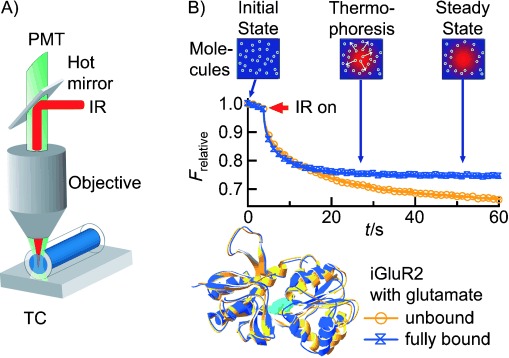
Label-free microscale thermophoresis. A) A capillary containing a protein sample with intrinsic tryptophan fluorescence is placed on a thermoelectric cooler (TC) providing a constant basis temperature. Fluorescence is excited with an UV LED and recorded with a photomultiplier tube (PMT). The solution inside the capillary is locally heated with an IR laser, which is coupled into the fluorescence microscope using an IR-reflecting “hot” mirror. B) The fluorescence of the heated spot is recorded, normalized, and plotted against time. After the IR laser is switched on at *t*=5 s, the fluorescence decreases as the temperature increases, and the fluorescent protein molecules move away from the heated spot because of thermophoresis. The unbound iGluR2 ligand-binding domain (yellow; PDB code 1FTO) shows stronger thermophoretic depletion than the complex with glutamate (blue; PDB code 1FTJ).^[7]^ This reflects the conformational change of the protein upon binding.

The MST setup consists of a fluorescence microscope with a 1480 nm infrared laser coupled into its optical path (Figure 1 A). The laser is focused into the capillaries containing the sample, where it creates a temperature gradient. Up to now the thermophoretic movement has been detected using a fluorescent tag attached to one of the binding partners (standard MST). To avoid the possible drawbacks of labeling we propose the use of intrinsic protein fluorescence. It is mostly caused by the aromatic amino acids phenylalanine, tyrosine, and tryptophan (Trp), with the latter being the dominant intrinsic fluorophore. We used an UV-light-emitting diode for fluorescence excitation and a photomultiplier tube (PMT) to record emission. Especially in the short-wavelength regime around 350 nm used for label-free MST, photon-counting PMTs are more sensitive than the CCD cameras employed for standard MST.

Examples of measured fluorescence signals from label-free MST are shown in Figure 1 B. After the temperature has increased, the fluorescence initially changes rapidly as an inherent property of the fluorophore. This “temperature jump”, which occurs on a timescale of 100 ms, can easily be distinguished from the subsequent rather slow thermodiffusion lasting several seconds.^[11]^ To infer binding affinity, a titration series is prepared in which the concentration of the ligand is varied while the concentration of the protein is kept constant. For each dilution step, the temperature perturbation is applied and the fluorescence response is recorded. The thermophoretic signal changes stepwise with increasing ligand concentration. This corresponds to the changing ratio of unbound protein to bound complex and reflects the alteration of molecular properties upon binding (Figure 1 B). To derive the dissociation constant *K*_D_ from the raw data, the fluorescence signals are normalized to the undisturbed situation before heating. Working with these relative signals avoids the difficulties of analyzing absolute fluorescence levels or small alterations in absorption and emission spectra upon binding. As known from standard protein fluorescence spectroscopy, such signals can be complex to interpret, mostly because of the presence of multiple Trp residues or energy transfer between amino acids.^[14]^ In the following examples, we prove that label-free MST is a valuable tool to study the binding of numerous types of ligands to different protein classes.

In the mammalian central nervous system iGluRs play a key role in fast excitatory synaptic transmission.^[15]^ The investigation of ligand binding to the various iGluR subtypes is in the focus of ongoing research.^[12]^ Using label-free MST we analyzed the interaction of the non-NMDA receptor subunits iGluR2 and iGluR6 with different agonists. We used soluble LBD versions generated by fusing the two discontinuous extracellular fragments S1 and S2.

The LBD of the AMPA receptor subunit iGluR2 (29.2 kDa; Figure 1 B) contains four tryptophan residues. A solution with a concentration of 2 μm exhibited sufficient UV fluorescence intensity without significant bleaching. Analyzing the change in thermophoretic mobility, we found a *K*_D_ value of (835±43) nm for the natural agonist glutamate (147.13 Da; Figure 2). This accurately reproduces the literature value of 821 nm.^[16]^ Azobenzene glutamate (glu-azo; 367.15 Da), a photoswitchable agonist allowing for remote control of neuronal excitability, binds to the iGluR2-LBD with a *K*_D_ value of (19±5) μm (Figure 2).^[17]^ MST confirms the finding that glu-azo, designed as a kainate receptor ligand, also binds iGluR2.^[18]^ Adding glu-azo to iGluR2-LBD preincubated with a saturating amount of glutamate (500 μm) did not influence thermophoresis. The result proves the specificity of the glu-azo signal and indicates that both agonists compete for the same binding site. We verified results of the label-free measurement for glu-azo by performing standard MST with labeled iGluR2-LBD (see the Supporting Information, Figure S-1). The measured *K*_D_ value of (22±8) μm does not deviate significantly from the value determined by the label-free analysis. This demonstrates that label-free MST was not disturbed by autofluorescence and that the label did not affect the interaction. We additionally quantified ligand binding to the kainite receptor subunit iGluR6. We used the iGluR6-LBD (4 Trp; 29.3 kDa) in a concentration of 2 μm. The determined upper limit of 359 nm for the affinity to glutamate is in agreement with the reported *K*_i_ value of (355±74) nm (see Figure S-2).^[19]^ The iGluR6-LBD binds glu-azo with a *K*_D_ value of (3.2±0.4) μm (see Figure S-3).

**Figure 2:**
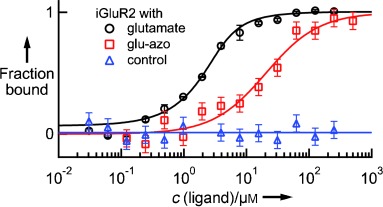
Ligand binding to membrane receptors. Binding curves are derived from the specific change in the thermophoretic mobility upon ligand titration to a constant iGluR2-LBD concentration of 2 μm. The curves show binding affinities of (835±43) nm for glutamate and (19±5) μm for glu-azo. The two agonists compete for the same binding site, as preincubation of iGluR2 with a saturating amount of glutamate prevents glu-azo binding (control).

Label-free MST is sensitive enough to measure the binding of small molecules. Three selective small-molecule inhibitors of p38α (59.5 kDa) were tested. P38 is a mitogen-activated protein kinase (MAP kinase) responding to stress. The isoform p38α is considered the key subtype involved in cytokine synthesis during inflammatory response. Thus, potent inhibitors of p38α might lead to the development of effective novel approaches for the treatment of inflammatory diseases.^[20]^

The kinase contains five Trp residues, so that a concentration of 100 nm was sufficient. As shown in Figure 3 p38α binds the inhibitor SB202190 (331 Da) with a K_D_ value of (48±21) nm, reproducing the literature value of 37 nm.^[21]^ The upper limits of affinity for PD169316 (360 Da) and SB239063 (368 Da) were determined to be 33 nm and 20 nm, respectively. This is in good agreement with reported IC_50_ values of 130 nm (PD169316) and 44 nm (SB239063).^[22,^^23]^ Nonspecific interactions can be excluded as thermally denatured kinase p38α did not show binding.

**Figure 3:**
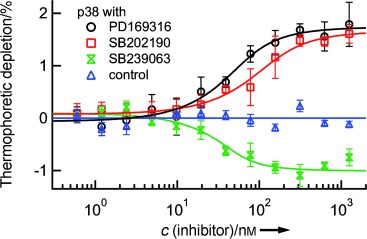
Screening of small-molecule kinase inhibitors. Three selective inhibitors were successfully tested for binding to the nonactivated form of MAP kinase p38α (*c*=100 nm). Corresponding to structural differences, the binding of SB202190 and PD169316 has the opposite effect on the thermophoretic movement compared to SB239063. SB202190 binds with a *K*_D_ value of (48±21) nm. The upper limits of affinity for PD169316 and SB239063 were determined as 33 nm and 20 nm, respectively. These results are in good agreement with previously reported values. Thermally denatured p38α did not show binding (control).

Remarkably, the thermophoretic signal contains further information on the ligands. The complexes formed from SB202190 (and PD169316) to p38α show less thermal depletion than the unbound kinase, which is represented by the positive slope of the binding curve. The binding of SB239063 has the opposite effect (Figure 3). Apart from a single functional group, the compounds 4-(4-fluorophenyl)-2-(4-hydroxyphenyl)-5-(4-pyridyl)-1*H*-imidazole (SB202190) and 4-(4-fluorophenyl)-2-(4-nitrophenyl)-5-(4-pyridyl)-1*H*-imidazole (PD169316) are identical in structure. The structure of the second-generation inhibitor SB239063 (*trans*-1-(4-hydroxycyclohexyl)-4-(4-fluorophenyl)-5-(2-methoxypyridimidin-4-yl)imidazole), however, differs significantly. These differences are likely to be the cause of the opposite effect on thermophoretic depletion.

Using label-free microscale thermophoresis, we successfully quantified different types of biomolecular binding events which are summarized in Table 1. Affinities reported in the literature were confirmed for all groups of interactions. For the LBD of the membrane receptor iGluR2, we observed that glu-azo binds to the same site as glutamate, yet with a much lower affinity. The affinity of small-molecule binding to the kinase p38α was measured and we found that structurally different inhibitors had an opposite influence on the thermophoretic depletion. This interesting finding suggests that thermophoresis could be used to not only determine binding strength but also gather additional information on the binding event. Comparative label-free MST studies would be necessary and might be a putative tool to classify novel ligands. The use of label-free MST is, however, not restricted to small-molecule testing. We also demonstrated the applicability for aptamer and ion binding (see the Supporting Information).

**Table 1:** Protein binding studied by label-free MST.[a]

Binding event	*K*_D_ values from label-free MST	Literature values
*iGluR2*		
glutamate	(835±43) nm	821 nm[16]
glu-azo	(19±5) μm	–
		
*iGluR6*		
glutamate	≤359 nm[b]	(355±74) nm (*K*_i_)[19]
glu-azo	(3.2±0.4) μm	–
		
*p38α*		
SB202190	(48±21) nm	37 nm[21]
PD169316	≤33 nm[b]	130 nm (IC_50_)[22]
SB239063	≤20 nm[b]	44 nm (IC_50_)[23]
		
*thrombin*[c]		
15 mer	(32±15) nm	25 to 100 nm^[25,^^26]^
29 mer	(133±42) nm	0.5 or 100 nm^[26,^^27]^
		
*Syt1*[c]		
Ca^2+^	(326±26) μm	50 μm to 3 mm[28]

[a] For all types of biomolecular binding events the measured affinities were in agreement with reported literature values.

[b] These affinities represent upper limits. The exact error estimations can be found in the Supporting Information.

[c] See the Supporting Information for descriptions, figures, and experimental details.

Label-free MST is particularly suitable for screening approaches as a typical interaction measurement requires only about 50 μL of a protein solution at a concentration of 0.1–2.0 μm. Furthermore, the measurement only takes about 5–10 min. This is made possible by the simple “mix-and-read” protocol without laborious sample preparations like surface immobilization or labeling. As the binding partners are not attached to a label or surface, molecular properties are not altered and mobility is not restricted. Thus native binding affinities are measured.

Label-free MST requires a sufficient intrinsic fluorescence signal of the protein, whereas difficulties arise from the UV fluorescence of the ligand and buffer. The UV fluorescence of the buffer, for example, caused by a Trp-containing spectator protein adds to background fluorescence leading to increased noise and a constant offset in the thermophoretic signal, but not to a change in the affinity specified by the binding. If both binding partners show a similar Trp fluorescence, direct quantification with label-free MST is not possible. The contribution of the titrated fluorescent ligand to the measured thermophoresis signal needs to be quantified by control experiments and then subtracted. This corrected thermophoresis signal should make it possible to infer the thermophoretic binding signal. However, most ligands, including the group of small molecules accounting for the majority of today’s pharmaceuticals do not exhibit UV fluorescence.

A protein of average Trp content (≥2 Trps) can be used in concentrations down to 100 nm, making it possible to accurately quantify *K*_D_≥50 nm. Interactions with higher affinities can still be detected qualitatively, but not precisely quantified. If insufficient Trp residues are incorporated, it is possible to introduce Trp by mutation. A conservative exchange of another aromatic amino acid for Trp often does not affect the molecular properties and protein function.[24] Alternatively, labeling and standard MST can be chosen, which typically can be used for lower protein concentrations and thus for the exact determination of affinities in the region of *K*_D_<1 nm.[9] Considering its practicability and applicability described above, label-free MST should be a promising novel tool to enhance knowledge on protein binding in all fields of life science.

## Experimental Section

The setup is based on a Zeiss Axiotech Vario microscope with a 40× quartz objective, numerical aperture 0.8 (Partek GmbH, Muenster, Germany). An UVTOP LED with a center wavelength of 285 nm (Thorlabs GmbH, Dachau, Germany) was used for excitation, a photomultiplier tube (P10PC, ET Enterprises Ltd, Uxbridge, UK) for detection. Fluorescence filters for tryptophan (F36-300) were purchased from AHF-Analysentechnik (Tuebingen, Germany). Fused-silica capillaries from Polymicro Technologies (Phoenix, USA) with different inner diameters and volumes of approximately 500 nL were used. Prior to the measurement, the polyimide coating was removed with an open flame and the capillaries were cleaned with ethanol on the outside. The temperature gradients were created with an IR laser diode (Furukawa FOL1405-RTV-617-1480, *l*=1480 nm, *k*=320 μm for water, 320 mW maximum power) purchased from AMS Technologies AG (Munich, Germany). The IR laser beam was coupled into the path of fluorescence light with an IR-reflecting “hot” mirror (NT46-386; Edmund Optics, Barrington, USA) and focused into the fluid with the microscope objective. As measured using the temperature-dependent fluorescence of TAMRA dye, the temperature in the solution was increased by 6 K in the beam center with a 1/e^2^ diameter of 25 μm. All measurements were performed at a capillary basis temperature of 20 °C. The capillary basis temperature was controlled with a thermoelectric cooler.

The expression vectors for iGluR2- and iGluR6-LBDs were kindly provided by Mark Mayer. P38α was provided by Krishna Saxena; PD169316, SB202190, and SB239063 were purchased from Sigma Aldrich (Munich, Germany). For the standard MST control, iGluR2-LBD was labeled using the Monolith NT Protein Labeling Kit RED according to the supplied protocol.

Measurements were conducted in the following buffers: iGluR2- and iGluR6-LBD: 10 mm HEPES pH 8.0, 150 mm NaCl, 1 mm EDTA; p38α: 50 mm Tris pH 7.8, 150 mm NaCl, 10 mm MgCl_2_, 0.05 % TWEEN20. As a control, p38α was incubated at 95 °C for 1 h. All solutions were incubated at room temperature for 1 h after the proteins had been mixed with the different target molecules.

The *K*_D_ values were obtained by fitting the fraction of bound proteins to the quadratic solution of the binding reaction equilibrium, derived from the law of mass action, with the *K*_D_ being the single free parameter.[29] The fitting function, number of repetitions, and the explanation of error bars are provided in the Supporting Information.

## Notes

Detailed facts of importance to specialist readers are published as ”Supporting Information”. Such documents are peer-reviewed, but not copy-edited or typeset. They are made available as submitted by the authors.
