# Supplementary material for: Label-Free Microscale Thermophoresis Discriminates Sites and Affinity of Protein–Ligand Binding
Source: Angew Chem Int Ed Engl. 2012 Sep 24;51(42):10656–9. doi: 10.1002/anie.201204268 (PMC3588113; doi:10.1002/anie.201204268)
Supplement: Supplementary file 1 [file anie0051-10656-sd1.pdf]

Angewandte Chemie
Eine Zeitschrift der Gesellschaft Deutscher Chemiker

Supporting Information
© Wiley-VCH 2012
69451 Weinheim, Germany

Label-Free Microscale Thermophoresis Discriminates Sites and Affinity of Protein–Ligand Binding**

*Susanne A. I. Seidel, Christoph J. Wienken, Sandra Geissler, Moran Jerabek-Willemsen, Stefan Duhr, Alwin Reiter, Dirk Trauner, Dieter Braun, and Philipp Baaske**

anie_201204268_sm_miscellaneous_information.pdf

Table of contents

1 *Fitting function*
2 *Number of repeats and error bars*
3 *Exact error estimations for values given as an upper limit*
4 *Additional iGluR binding curves*
5 *Additional experiments*
  5.1 *Aptamer binding to thrombin*
  5.2 *Ca^{2+} -ion-binding to synaptotagmin 1*
  5.3 *Additional experimental section*

1 Fitting function

To determine the affinity of a ligand L binding to a protein P via label free MST, the total concentration of the ligand c_{L0} is titrated, while the total concentration of the protein c_{P0} is kept constant. For the binding event of L to P, the mass action law reads

$$K_D = \frac{c_L c_P}{c_{LP}} = \frac{(c_{L0} - c_{LP})(c_{P0} - c_{LP})}{c_{LP}}$$

with

K_D : dissociation constant
c_L : free ligand concentration
c_P : free protein concentration
c_{LP} : concentration of the bound complex
c_{L0} : total concentration of the ligand and
c_{P0} : total concentration of the protein.

Solving for the fraction of occupied binder B yields

$$x = \frac{c_{LP}}{c_{P0}} = \frac{c_{L0} + c_{P0} + K_D - \sqrt{(c_{L0} + c_{P0} + K_D)^2 - 4c_{L0}c_{P0}}}{2c_{L0}}.$$

2 Number of repeats and error bars

The number of independent repeats was at least 3 for all measurements except for syt1 and iGluR6 (n=2); error bars show the standard deviation between these independent repeats. For iGluR2 binding glu-azo and the control with the saturated amount of glutamate and with the labeled protein n=1. In these cases the error was estimated by the noise of the fluorescence detection.

3 Exact error estimations for values given as an upper limit

The fitting procedure assumes a Gaussian, symmetric error distribution. Due to this approximation, the distribution reached negative values in three cases. The corresponding values are given as upper limits of affinity in the manuscript. The exact error estimations are listed in Table S-1.

Table S-1. In three cases an upper limit of affinity is given. The exact error estimations are listed below.

Binding event	Upper limit given in manuscript	Exact values derived from data fit
iGluR6		
glutamate	≤359 nM[a]	153±206 nM
p38α		
PD169316	≤33 nM[a]	18±15
SB239063	≤20 nM[a]	8±12

4 Additional iGluR binding curves

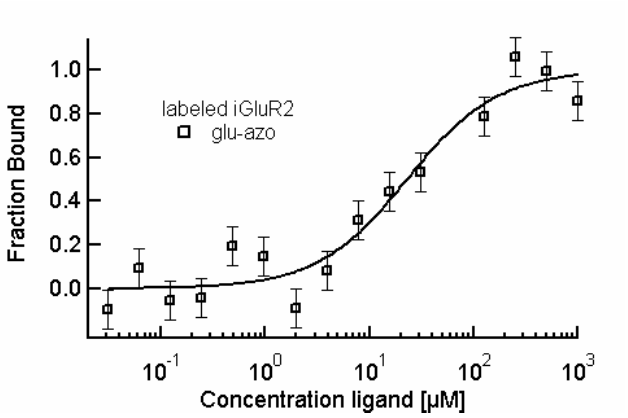

Figure S-1. Standard MST control for the binding of the iGluR2-LBD to glu-azo. The protein was fluorescently labeled and used in a concentration of 200 nM. The measured K_D of 22±8. µM verifies the label free result (19±5 µM) thus excluding disturbances by autofluorescence.

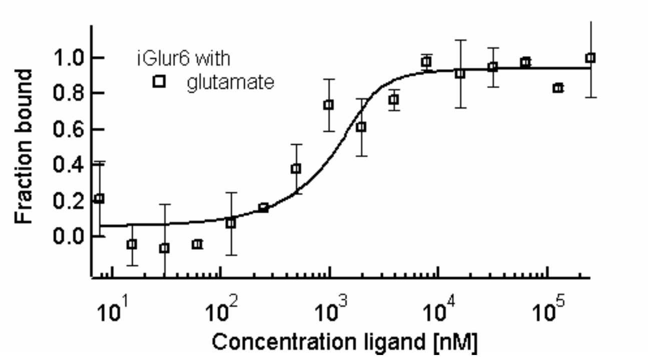

Figure S-2. Label free MST measurement of glutamate binding the iGluR6-LBD (c=2 µM). An upper limit of affinity of 359 nM was derived.

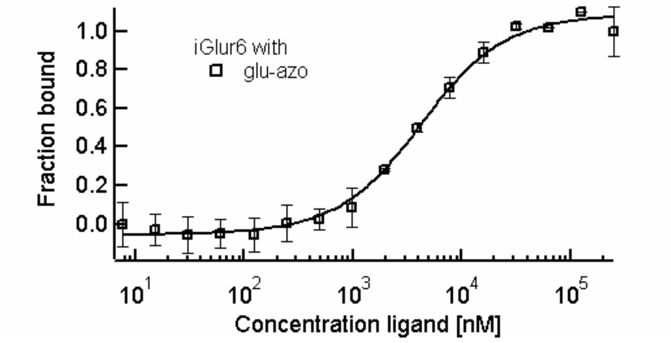

Figure S-3. Label free MST measurement of glu-azo binding the iGluR6-LBD (c=2 µM). The determined K_D is 3.2±0.4 µM.

5 Additional experiments

5.1 Aptamer binding to thrombin

Despite their much simpler production and higher stability, DNA aptamers resemble the binding behavior of antibodies. We demonstrate the applicability of label-free MST to monitor the binding of these valuable molecular tools using the example of human α-thrombin (36.7 kDa; 9 Trp residues). This serine protease is part of the human coagulation cascade. In 1992 Bock et al. designed a single stranded 15mer DNA aptamer binding to the fibrinogen recognition exosite.[1] Five years later Tasset et al. reported on a 29mer binding to the heparin exosite.[2]

For our label-free MST analysis of these aptamers, we used a constant thrombin concentration of 200 nM (Fig. S-4). The measured K_D of 32±15 nM for the 15mer reproduces the previously reported standard MST result with 5'-fluorescently labeled 15mer (K_D=30±19 nM).[3] It is in good agreement with literature values specifying the affinity as 25 to 100 nM based on filter binding assays.[1,2,4] For the 29mer, label-free MST reported an affinity of 133±42 nM. This is much higher than the K_D of 0.5 nM, reported by Tasset et al.[2] However, SPR measurements could not confirm the very high affinity of the 29mer either and instead reported a K_D in the range of 100 nM.[5] Tang et al. even found an affinity lower than for the 15mer consistent with our results.[6] Apart from the specific aptamers a 15mer dinucleotide mutant was measured. The mutant did not show binding, thus proving specificity.

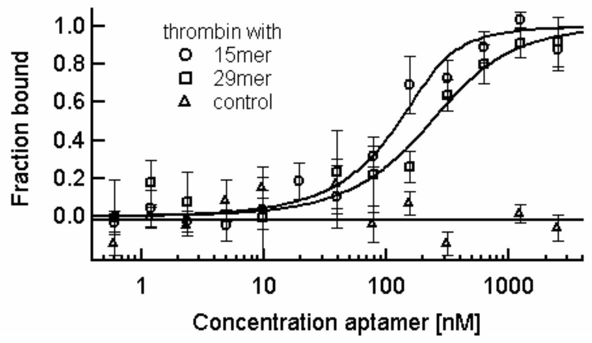

Figure S-4. Thrombin interaction with aptamers. Specific 15mer and 29mer DNA aptamers bind to the fibrinogen and heparin exosites of human α-thrombin (c=200 nM). We measured a K_D of 32±15 nM for the 15mer and a K_D of 133±42 nM for the 29mer and. A 15mer dinucleotide mutant did not bind to thrombin (control).

5.2 Ca^{2+} -ion-binding to synaptotagmin 1

As previously reported protein binding to ions can be measured with standard MST.[7] This type of binding events is also accessible via label-free MST. We analyzed the interaction of the synaptic vesicle protein synaptotagmin 1 (Syt1) with Ca^{2+}. The cytoplasmic C-terminal part of the protein consists of two C2 domains, C2A and C2B. NMR suggests that three and two Ca^{2+} bind to C2A and C2B, respectively. Upon binding, the C2 domains mediate membrane translocation. Hence synaptotagmin can act as a neuronal Ca^{2+} sensor triggering exocytosis of neurotransmitters into the synaptic cleft.[8]

We used Syt1's C2AB fragment in a constant concentration of 1 µM and titrated $CaCl_2$. Label-free MST yielded an overall Ca^{2+} affinity of 326±26 µM (Fig. S-5). A standard MST control with fluorescently labeled C2AB confirmed this affinity (see Supporting information Fig. S-6). Our results are in good agreement with ITC measurements by Radhakrishnan et al.[9] Assuming quintuple binding, the group extracted K_Ds of 48, 142 and 3120 µM for C2A and 490 µM for both Ca^{2+} binding to C2B. To obtain distinct K_Ds for the different binding sites, MST measurements of selective Ca^{2+} binding mutants of C2AB seem to be very suitable.[10] Titrating $MgCl_2$ did not result in a change in thermophoresis, demonstrating the specificity of the label free MST analysis (Fig. S-5). Standard MST experiments on cooperative Ca^{2+} and phosphatidylinositol 4,5-bisphosphate binding to Syt1 have already been performed successfully, suggesting that label free MST could also be used for extensive studies of Syt1 function.[11]

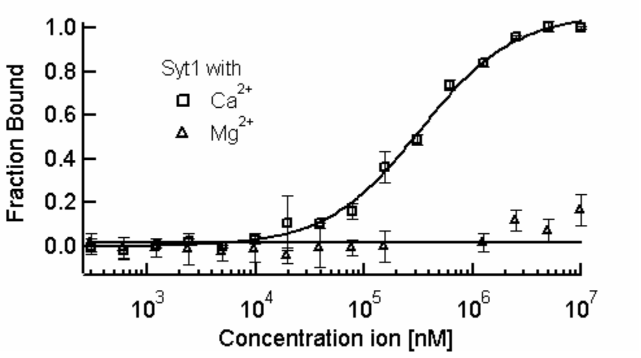

Figure S-5. Synaptotagmin 1 binds Ca^{2+} ions. Syt1's C2AB fragment was used in a constant concentration of 1 µM. $CaCl_2$ titration alters the thermophoretic mobility. An overall binding affinity of 326±26 µM was derived. In contrast, $MgCl_2$ does not have an influence on thermophoresis, documenting specificity.

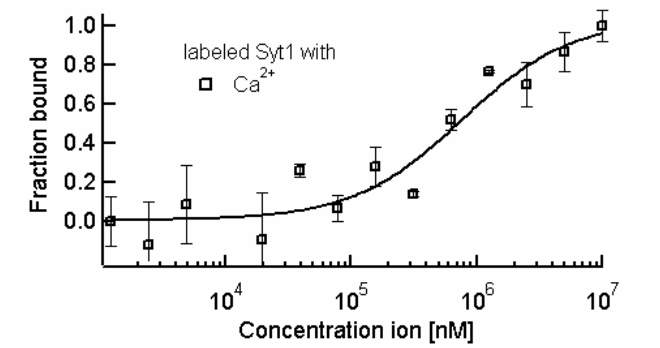

Figure S-6. Standard MST control for the binding of Syt1-C2AB to glu-azo. The protein was fluorescently labeled and used in a concentration of 40 nM. The measured K_D of 757±299. μM verifies the label free result (326±26 μM) thus excluding disturbances by autofluorescence.

5.3 Additional experimental section

Human α-thrombin was purchased from Haematologic Technologies Inc. (Essex Junction, USA). DNA oligonucleotides were synthesized by Metabion (Martinsried, Germany). The sequences of the oligonucleotides, with mutations as small letters, are: 15mer 5’-GGT TGG TGT GGT TGG-3’; 29mer: 5’-AGT CCG TGG TAG GGC AGG TTG GGG TGA CT-3’;f 15mer dinucleotide mutant: 5’-GGT TGt TGT GGT TtG-3’. The aptamers were denatured and re-natured prior to the experiments to ensure that they reached their active conformation. The Syt1 C2AB construct from *R. norvegicus* was cloned into a pET28a expression vector. The purified protein was kindly provided by Geert van den Boogaart.

For the standard MST control Syt1-C2AB was labeled using the Monolith NT Protein Labeling Kit RED according to the supplied protocol.

Measurements were conducted in the following buffers: Thrombin selection buffer: 20 mM Tris-HCl pH *7.4*, 150 mM NaCl, 5 mM KCl, 1 mM $CaCl_2$,1 mM $MgCl_2$, 0.1% TWEEN20;[1] Syt1: 20 mM HEPES pH *7.4*, 150 mM KCl, for the labeled control additionally 0.5 mg/ml BSA. All solutions were incubated at RT for 1h after the proteins had been mixed with the different target molecules.

[1] L. C. Bock, L. C. Griffin, J. A. Latham, E. H. Vermaas, J. J. Toole, *Nature* **1992**, *355*, 564–566.

[2] D. M. Tasset, M. F. Kubik, W. Steiner, *J Mol Biol* **1997**, *272*, 688–698.

[3] P. Baaske, C. J. Wienken, P. Reineck, S. Duhr, D. Braun, *Angew. Chem. Int. Ed. Engl.* **2010**, *49*, 2238–2241.

[4] M. C. R. Buff, F. Schäfer, B. Wulffen, J. Müller, B. Pötzsch, A. Heckel, G. Mayer, *Nucleic Acids Res.* **2010**, *38*, 2111–2118.

[5] P.-H. Lin, R.-H. Chen, C.-H. Lee, Y. Chang, C.-S. Chen, W.-Y. Chen, *Colloids Surf B Biointerfaces* **2011**, *88*, 552–558.

[6] Q. Tang, X. Su, K. P. Loh, *J Colloid Interface Sci* **2007**, *315*, 99–106.

[7] C. J. Wienken, P. Baaske, U. Rothbauer, D. Braun, S. Duhr, *Nat Comms* **2010**, *1*, 100.

[8] H. Tokuoka, Y. Goda, *Neuron* **2003**, *38*, 521–524.

[9] A. Radhakrishnan, A. Stein, R. Jahn, D. Fasshauer, *Journal of Biological Chemistry* **2009**, *284*, 25749–25760.

[10] A. Stein, A. Radhakrishnan, D. Riedel, D. Fasshauer, R. Jahn, *Nat. Struct. Mol. Biol.* **2007**, *14*, 904–911.

[11] G. van den Bogaart, K. Meyenberg, U. Diederichsen, R. Jahn, *Journal of Biological Chemistry* **2012**, *287*, 16447–16453.
